# Stakeholders' Actions, Responsibility and Limitations in Support of Nursing Students Experiencing Workplace Violence During Clinical Placement: The Clinical Facilitators View

**DOI:** 10.1111/jocn.17706

**Published:** 2025-02-19

**Authors:** Hila Ariela Dafny, Nicole Snaith, Paul Cooper, Nasreena Waheed, Stephanie Champion, Christine Mccloud

**Affiliations:** ^1^ College of Nursing and Health Sciences Flinders University Bedford Park South Australia Australia; ^2^ Caring Futures Institute Flinders University Bedford Park South Australia Australia; ^3^ College of Nursing and Health Sciences Adelaide South Australia Australia

**Keywords:** clinical supervision, mental health, nursing students, physical abuse, preceptorship, workplace violence

## Abstract

**Background:**

Workplace violence toward nurses is a significant global issue affecting their mental and physical health, job satisfaction and performance, and can ultimately lead to decisions to leave the profession. As the least experienced caregivers in the health workforce, nursing students are particularly vulnerable to experiencing workplace violence and are often powerless to deal with WPV incidents.

**Aim:**

To examine clinical facilitators' insights into how to support nursing students following experiences of workplace violence during their clinical placement.

**Design:**

An exploratory, descriptive qualitative design.

**Methods:**

Data were collected between September and November 2022 using semi‐structured interviews with 11 clinical facilitators working in South Australia, each lasting about 1 h. The interviews were transcribed verbatim and analysed using thematic analysis.

**Results:**

Clinical facilitators identified that many students found support and solace from avenues outside of the CFs and university staff, including ward staff, family, friends and other students. However, students are limitedly prepared for the realities of clinical work, particularly concerning workplace violence, and that the university supports available were reactive to events in the clinical environment.

**Conclusion:**

Addressing workplace violence requires systemic changes, better support for clinical facilitators and a steadfast commitment by all stakeholders to student safety.

**Implications for the Profession:**

Solid collaborations between universities and clinical facilities with clear guidelines and direct lines to address potential violence issues are essential. Zero‐tolerance policies regarding workplace violence could provide a safer environment that promotes nursing student learning outcomes, safer placements, better student experiences and optimal healthcare provision.

**Reporting Method:**

COREQ guidelines were adhered to for reporting qualitative research.

**No Patient or Public Contribution:**

This paper specifically explores the perspective of the clinical facilitator's experience of WPV in their role of supporting student learning during clinical placement.

**Summary**

- The study addressed the clinical facilitators' views of the available support provided to nursing students following experiences of workplace violence during their clinical placement.
- Clinical facilitators felt limited in their role to support students experiencing workplace violence and that a proactive and collaborative approach is required to prepare nursing students prior to their clinical placements.
- This research impacts future nursing students, education providers, clinical facilitators and clinical placement providers.

## 1 Introduction

Registered nurse training has, for many decades in Australia, been located in university vocational course programs which include a designated time and duration of clinical placement. It is during this time that registered nurse students (RNS) spend quarantined learning in a clinical venue, honing their skills, knowledge and experience through exposure to the real world of health care. The clinical placement experience is a contractual arrangement between a university and a clinical venue, wherein the venue and the university agree to provide support for RNS in achieving learning outcomes. It is, however, in these clinical venues where RNS are increasingly being exposed to and enduring workplace violence (WPV) and where support is often found to be lacking. More than 42% of student nurses describe experiences of WPV during clinical placement (Tee and Valiee 2020; Dafny and Muller 2021). WPV is often complex, and may be subtle events that can include racism, incivility, sexual or psychological abuse, and direct physical abuses (Tee and Valiee 2020; Dafny and Muller 2021). Perpetrators of WPV can include patients, relatives, nursing staff, students and other clinical staff Liu et al. (2019). Support for RNS exposed to WPV is a broad concept that encompasses both proactive Heckemann et al. (2015) and reactive aspects Zhang et al. (2021) when managing WPV events. Proactive support is in the preparation of students for WPV via education, knowledge and skill development, whereas reactive support can be considered the many reporting systems and access to mental, emotional and physical health support programs following a WPV event Zhang et al. (2021). While the role of clinical facilitator (CF) is considered essential for enhancing student success in placement, the lack of role clarity, limited support and training for CFs, along with the cost‐cutting measures of universities, results in facilitators who are under pressure and caught between the expectations of universities and clinical settings Ryan and McAllister (2021; Hughes et al. 2023). The CF is an experienced registered nurse usually appointed by the university and is on site in venues to advocate and support the RNS Needham (2016). They are in a unique position to witness and assess the support provided to RNS by universities and venues following WPV events. This study mines the experiences of CFs in the support provided to RNS following WPV events.

## 2 Background

WPV toward nurses is a significant global issue affecting their mental and physical health, job satisfaction and performance, and can ultimately lead to decisions to leave the profession (Dafny and Beccaria 2020). Nursing students are particularly vulnerable to experiencing WPV (Birks et al. 2017; Budden et al. 2017) and feel powerless to deal with WPV incidents (ICN 2017). WPV impacts nursing students' physical and mental health, inhibits their learning experience, preventing them from developing appropriate caring behaviours with patients (Birks et al. 2017) and is associated with poor retention of students (Budden et al. 2017; Tee et al. 2016).

The 2017 International Council of Nurses position statement on the prevention and management of WPV defines WPV as involving ‘incidents where staff are abused, threatened or assaulted in circumstances related to their work, including commuting to and from work, involving an explicit or implicit challenge to their safety, well‐being or health’ ICN (2017). WPV may include physical assault, sexual assault and non‐physical violence such as verbal abuse, racism, bullying, hostility, incivility, intimidation or threats (Tee and Valiee 2020; Dafny and Muller 2021).

Developing effective strategies to prevent and manage WPV is essential to the future of the nursing profession. Globally, the literature highlights the need for specific training for nursing students in the curriculum and simulation of WPV to teach appropriate responses (Sanner‐Stiehr and Ward‐Smith 2017) and training and supervision for clinicians to safely work with RNS (Tee and Valiee 2020). Universities are viewed as largely responsible for ensuring that students are allocated to clinical settings that are safe and welcoming for students (Tee and Valiee 2020; Warshawski 2021). However, the literature highlights that universities and clinical settings should work in partnership to proactively create a student‐friendly culture and prepare and support staff rather than avoid certain placement settings or adopt a reactive approach to WPV (Tee et al. 2016). This includes specific training for both nurses and supervisors to prevent WPV toward nursing students and create a zero‐tolerance culture (Tee et al. 2016).

Lack of reporting of WPV by RNS is a significant issue highlighted in the literature. Some of the reasons include fear of negative influence on their grades and future employment (Birks et al. 2017; Kim et al. 2018), risk of further hostility from clinical staff (Uzar‐Ozecetin et al. 2021), repercussions from the university (Coutrney‐Pratt et al. 2018), being seen as less competent (Kim et al. 2018) and not being believed (Birks et al. 2017; Warshawski 2021). Challenges with the administrative processes of reporting are another barrier to reporting. The actual process of reporting WPV was viewed as a difficult and mentally tiring process by nursing and medical students in a study undertaken by Warshawski (2021). Students did not feel supported by the organisation in reporting due to the long and complex process and barriers put in place by the system. The under‐reporting of WPV is a barrier to implementing supports to address this significant issue. Tee et al. (2016) emphasised the need to raise awareness for students to identify WPV and its effects, understand that it is not part of the job, and empower them to report and know what supports are available when they do report.

Debriefing is identified as an important step in the process of supporting students and managing WPV. Nursing students in a study undertaken in Australia identified debriefing as important in better understanding and managing WPV (Courtney‐Pratt et al. 2018). However, some students were reluctant to be involved in debriefing as they did not want to cause issues and were unsure of the consequences. Courtney‐Pratt et al. (2018) emphasised that a skilled facilitator should be able to facilitate a debrief that is safe and inclusive and encourages active learning, but this can be difficult to achieve depending on skills, experience and confidence in the role. Students have also reported unsatisfactory responses from tutors, instructors and mentors from their university when they have reached out to debrief following WPV (Birks et al. 2017; Tee and Valiee 2020; Üzar‐Özçetin et al. 2021). Informal supports through peers, friends and family are often more commonly sought by nursing students (Dafny et al. 2023). This suggests a lack of confidence in the university and/or clinical placement to adequately support students following WPV, and prevent future WPV, which in turn may be another barrier to RNS reporting WPV.

CFs are in an ideal position to support RNS following WPV, including insights into how to prevent and manage future incidents. However, CFs are not always provided with clear direction and an outline of the expected role in supporting students on clinical placements; where facilitators are employed casually, there is a risk that they are not provided with the appropriate education and tools to support students in a challenging environment. Often, CFs are supporting students in environments where they are visitors themselves and are not familiar with the nuances of the culture they are entering.

## 3 The Study

Research aim: First, to examine CFs' insights into their role and preparedness in supporting nursing students following a WPV event during clinical placement. Second, to explore CFs' perceptions of universities and clinical venues' preparation for nursing students in WPV management in the clinical environment.

## 4 Methods

### 4.1 Design and Theoretical Framework

To answer the research questions, an exploratory, qualitative study design was seen as suitable. A semi‐structured interview guide was used to interview 11 CFs, and the data was analysed using thematic analysis (Braun and Clarke 2021). The open‐ended questions sought to explore the CFs' experiences and perceptions, providing valuable insights to the research questions.

### 4.2 Study Setting and Recruitment

Participants were recruited through purposive sampling from a metropolitan city in Australia. Consideration was given in seeking ‘information‐rich’ participants who have experience in supervising nursing students in a clinical setting and are willing to participate in the study. As such, snowballing was also used to recruit participants. This sampling method is consistent with the research design. Information packages were sent to prospective participants via email that included a brief description of the study and a return email address if they agreed to participate in the study or needed to contact the researchers to discuss any concerns they may have. Participants were required to have been a CFs within the last 12 months. Participants received a $30 voucher for their time.

In this qualitative analysis, it is important to acknowledge the biases and backgrounds of the research team. The team includes one male and five females, with five members having nursing experience (including as CFs), and two members having backgrounds in public health. All team members are experienced in qualitative research and conducting interviews, and four have worked previously on studies of WPV within healthcare settings. None of the research team had prior relationships with the interviewees. Before data collection, interviewers shared their field experiences within the team, and the possible impact of these preconceptions on their approach was considered and accounted for. The diversity of backgrounds was a benefit to the project, as researchers with nursing backgrounds brought insight, sensitivity and understanding to the interviews, while those without such backgrounds provided an objective perspective, supporting a balanced interpretation of the data.

### 4.3 Inclusion and Exclusion Criteria

The inclusion criteria required participants to be CFs employed in Metropolitan Adelaide, South Australia, either as casual or permanent staff. They needed to supervise undergraduate nursing students, regardless of the students' year level. Additionally, participants must have worked as CFs within the past 12 months. This study excluded CFs who have not practised within the last 12 months, those who supervised postgraduate nursing students, or CFs of other qualified health professionals, not particularly nursing.

### 4.4 Data Collection

The prospective participants were provided with a participant information sheet outlining the aim of the study, and a consent form, including a personalised explanation of the project and the interview process. They were assured that they can withdraw their consent at any time during the interview without fear of any impact on their engagement with the university. Participants who agreed to be interviewed chose their preferred location, face‐to‐face or via Microsoft Teams (online), date and time. The interviews were held outside the hospital or the clinical settings to provide a comfortable environment for the participants. Informed consent was obtained before the start of the interview and before recording. Demographic data was obtained at the beginning of each interview that lasted approximately 60 min. A semi‐structured interview guide was used in all interviews, allowing for the exploration of different issues in each interview. An outline of the questions asked in the interview included: Have you ever experienced or witnessed WPV while you were in clinical placement with nursing students? Would you like to share your experience? Do you think that nursing students are provided support after violent incidents? What kind of support? By whom? What do you suggest in order to reduce or avoid violence toward nursing students? As a CF? University? Your workplace/institution? These questions were used to guide the conversation rather than limiting it. Further open‐ended questions encouraged narrative answers from the participants. When necessary, probing and prompts such: ‘What do you mean by…?’ or ‘Please, tell me more about…’ were used to deepen the richness of the data collected.

Interviews were conducted until data saturation was achieved. The small sample size was not an issue as saturation could be achieved with small sample sizes (9–17 interviews) in homogenous study populations with narrow objectives (Hennink and Kaiser 2022), such as this study. A qualitative sample can be as small as a single person and sampling continues only until saturation is achieved (Shorten and Moorley 2014). All interviews were recorded and transcribed verbatim. The semi‐structured interviews (*n* = 11) were held between September and November 2022. The COREQ research‐reporting checklist based on Tong et al. (2008) was used to report the study.

### 4.5 Data Analysis

The data collected through individual semi‐structured interviews provided valuable insights to answer the research questions: How do CFs perceive their role and level of preparedness in supporting nursing students after experiencing WPV during clinical placements? What are CFs' views on the adequacy of university and clinical venue preparation for nursing students in managing WPV in the clinical environment? The interviews were transcribed into a Word document and analysed using Braun and Clarke's (2021) thematic approach. This approach included 6 steps: (1) Transcribing, reading, re‐reading and noting down the first impressions; (2) grouping common ideas into codes; (3) collating the codes and assigning them to initial themes; (4) creating a thematic map and ensuring the correct fit of codes and data to the themes; (5) refining and renaming the themes to ensure accuracy; and (6) incorporating the themes into the report (Braun and Clarke 2021). Thematic analysis was initially done by two of the research team members, followed by review and feedback by all the members of the research team for accuracy. The QSR NVivo 12 (QSR International 2021) software was utilised in the initial coding processes.

### 4.6 Ethical Considerations

Ethics approval to conduct the study was obtained from the authors' university Human Research Ethics Committee (approval number 5497) in 2022 before the study was conducted. Participation was voluntary, and informed consent was obtained prior to the interview and dissemination of results.

### 4.7 Rigour and Reflexivity

Methodological rigour ensures the quality of the study and minimises biases. The research team ensured purposive sampling of CFs and explained in detail the study process, including data collection and analysis, to improve the quality, validity and reliability of the study findings. Overall rigour refers to the study's consistency and transparency and ensures that the research findings' dependability, confirmability and transferability occur. The research team described the research process and provided the findings accurately by providing the participants' quotes and accurately reflecting their perceptions. Investigator triangulation, as described by Polit and Beck (2017) was a significant feature of the analysis process to ensure that bias and idiosyncratic interpretations were avoided.

Reflexivity increases the quality of the research because the research team's understanding of their positions and interests might impact the research process. The research team acknowledged that most members were experienced registered nurses currently working as nursing lecturers and researchers. Their familiarity with nursing studies and clinical placements could influence the study. To minimise potential bias, two team members conducted an independent thematic analysis of the data. They reached a consensus on the themes and held multiple discussions about the quotes to ensure the participants' meanings were accurately captured.

## 5 Results

### 5.1 Characteristics of Participants

The participants (*n* = 11) ranged in age from 32 to 61 years 63.6% (*n* = 7) of whom were females. While the participants came from multiple backgrounds, the main cultural background was Australian, 54% (*n* = 6), African (*n* = 2), Chinese (*n* = 1), UK (*N* = 1) and one did not mention. The highest educational qualification achieved varied, with 54% (*n* = 6) participants having completed an Advanced Diploma, and 27% (*n* = 3) a Master's degree, and having had additional training in many specialties, including oncology, cardiac nursing, critical care nursing, emergency nursing, mental health nursing, clinical education, paediatrics and midwifery. Their experience as RNs ranged from 5 to 38 years, with an average of 18.5 years and an average of 9.5 years of clinical supervision experience. Most CFs worked part‐time, 54% (*n* = 6) and on average worked 14.7 h per week as CFs.

Three major themes found include the following: (1) CFs: Role, actions and experiences; (2) University: Reactive support and limitations; and (3) Clinical venue: Support actions and limitations. Major themes and subthemes as identified in Table 1 are discussed in detail below.

**TABLE 1:** Overarching theme, major themes and subthemes identified through thematic analysis.

‘Stakeholders actions, responsibility and limitations in support of registered nursing students experiencing workplace violence during clinical placement: The clinical facilitators view’	
Major themes	Subthemes
Workplace violence support of student registered nurses; clinical facilitators, actions and experiences	Clinical facilitator support actions
	Recognising support from others
	Future proofing for WPV by clinical facilitators
University: Reactive support and limitations	WPV, Policies, processes and services
	Disparity between University and Clinical venue understanding of role and learning outcomes of RNS placement
	Limited preparedness of RNSs for WPV
	Lack of support for RNS
Clinical venue: Support actions and limitations	Effect of clinical allocation
	Active role and support by clinical venue
	Positive ward experiences
	Limitation of support following WPV event at clinical venues

### 5.2 CFs Actions and Experiences

The first major theme of this study explores the thoughts, actions and perceptions of the CFs, with a focus on WPV preparation, knowledge, events and support actions. As the stakeholder most closely aligned with RNS during placement, CFs have unique insight into WPV experiences of RNS, support required, and where limitations exist.

#### 5.2.1 CF Support Actions

Support for RNS by CFs was described in multiple ways and actions, including the importance of RNS knowing who to contact should a WPV event occur. CFs supported RNS by being accessible and available to their students. Most CFs provided their phone numbers to their students so they could be contacted after hours if something happened during their placement. Other CFs described holding regular meetings with their students in the placements so that students feel like part of the team and will be supported. Actions such as allowing an RNS to leave a shift early in recognition of the trauma of an event or directing RNS to support services and creating a collegial team were provided as evidence of support for RNS following a WPV event.

I always ask any of the nurses on the ward that if anything's occurred to let me know. I always make sure the students have my personal phone numbers and emails so they can contact me and talk to me immediately if anything occurs. So, I'd be the first point to go to. PARTICIPANT 8

They should know that they have a wide range of support so like the employee assistance programme, EAP, which the students have as health and counselling at uni. PARTICIPANT 7

I think creating a team environment, so meeting as a team at the very beginning and then at least once a week, means that they all know who's who, and if they need to change a shift for whatever reason they know that person. PARTICIPANT 6

Sometimes I guess the support is to actually give the students a break and give them an extended lunch break or maybe even get them to not, to finish their shift early because it's been such a big challenge for them. PARTICIPANT 5

Directing RNS to the available support services such as a helpline, lifeline, referrals to mental health services and services that can randomly check students was considered an important aspect of supporting RNS who had experienced a WPV event by CFs.

The only thing would be the social support, you know, if they need to call the helpline, the Lifeline sort of thing as well. PARTICIPANT 11

We can offer them the counselling services, keep briefing with them, inform the university, doing lots of checks at random times. PARTICIPANT 9

#### 5.2.2 Recognising Support From Others

Discussions by CF's concerning students who had experiences of WPV identified that many students found support and solace from other avenues including, ward staff, family, friends' and other students.

…mostly, the people that were the most supportive to her, or that she seemed to be the happiest to talk to is her family, so of course they were in another country. PARTICIPANT 11

I found that depending on the students themselves, they may have debriefed with the nurse that they were working with before I got to the situation, or they might actually be waiting for me or would have given me a call to say I need to talk to you. PARTICIPANT 7

The ward staff can be quite helpful sometimes, as well, after pretty big incidents, they do have their debriefs, and depending on the manager, some of them can be really supportive as well. PARTICIPANT 3

I've actually walked in and seen a receptionist giving a student a pat on the back because it was a very traumatic code black that went down. The student was in tears. So, there's that kind of humanity, I guess, in supporting people as well. PARTICIPANT 5

#### 5.2.3 Future Proofing for WPV by CFs

Prior to placements commencing, CFs described the importance of preparing the students for the individual wards by exploring students' knowledge, learning opportunities and explaining the type of patients and possible situations, including incidents of WPV. Most CFs felt that it was important to alert the RNS to potential WPV experiences and situations where violence could escalate, providing protective knowledge and understanding of antecedents to violence but also knowledge of where and when to report WPV events. CFs used preparatory time to provide students with an awareness of supports available to them following WPV events.

If they were starting placement in ED or a medical ward, it's being aware of the type of patients that they might find in those wards and then letting them know so these are the type of presentations that they might, might not be. It's just then creating that awareness for them. So, this type of work can happen. So, a lot of education from me about violence, about the type of patients that are presenting and what they can expect. That might be, some students might find that to be true or some students might say ‘Actually I didn't get to experience what we've talked about but at least I know that I've discussed it with them’. PARTICIPANT 7

If we, we understand that this sort of thing can happen, then we can forewarn the students and help them understand this thing. And help them understand the processes for reporting that stuff back, if they need to… we've got plenty of supports for the students if they feel negatively impacted by any of those. I certainly think we've got those processes in place… Lots of mentor supports, we have medical services we can refer onto if we need to, if things are that bad. Mental health support, financial support, if things have progressed through violence that it's an impacted a student financially, which it could do. We can then, we can support with that as well. PARTICIPANT 1

Despite CFs' extensive knowledge of services available many felt that universities should provide further education to the CFs on what the students are studying regarding WPV in the placements. In addition, further education should be provided to RNS by teaching them different scenarios or facilitator workshops as part of their competencies, including what process they should follow, and ways to better support nursing students.

It would be nice for us to have a bit of a workshop as well and learn what the university has told the student. A bit like when we go into the laboratories, we learn what the students are being taught so that when they go on placement and if they say, no they can't do something like that, we'll say well we did do it in the laboratory you know. I think new could do a bit of a workshop whether we incorporate that into the facilitator workshop… because obviously we don't get a lot of facilitators necessarily turn up to all of those, whether it be an annual update that facilitators do, yeah part of your competencies or something. PARTICIPANT 9

The actions and personal supports provided by CFs were localised to the clinical placement arena and were both proactive and reactive to potential and actual WPV events during clinical placement. Most CFs described the support that the university provided to students following WPV events but were often critical of the university preparation of RNS for clinical placement. The supports provided by the university, and limitations of these supports during WPV encounters are described in the second major theme of this study.

### 5.3 University Reactive Support and Limitations

All RNS who present to clinical sites for practical experiences have been through at least the first year of undergraduate nursing studies where knowledge and practical skills have been presented. CFs were able to describe the policies and procedures designed to support RNS during clinical placement; however, in the opinion of the CFs, there was limited preparation of students for the realities of clinical work, particularly in relation to WPV, and that university supports available were reactive to events rather than proactive preparation of RNS for WPV in the clinical environment.

#### 5.3.1 WPV, Policies, Processes and Services

Universities support nursing students in clinical placement by teaching clinical skills and providing services such as tutors, lectures, facilitations and other administrative support. In addition, universities provide clinical education teams to support students and, if needed, counselling services and social workers' support services or assistance in moving a student to a different placement. The CFs were aware of the programs and formal avenues provided by universities, and RNS could be directed to these services following WPV events.

But also, we have a reporting channel where you can lodge in an incident such as SLS. And then we also have a clinical education team that are basically there to support the students. PARTICIPANT 10

There probably is a lot of stuff around, like I would say the student learning and … and things like that have got information about self‐care and keeping aware of your own limitations and checking in on your own mental health and things like that, which is probably a form of a strategy to assist students. PARTICIPANT 5

#### 5.3.2 Disparity Between University and Clinical Venue Understanding of Role and Learning Outcomes of RNS Placement

One issue that CFs identified as potentially contributing to WPV was a lack of understanding of the RNS role and responsibilities provided by the University to Clinical venues and nursing staff. CFs thought this could be achieved first by defining the CFs' and RNS roles and providing further information to nurses about what the CFs and RNS' can do safely within their roles and responsibilities.

And I think the university, so that bridge needs to be gapped by further information given to the people on the floor, the clinicians that are actually working side by side with these student nurses, every area, they all need to be, the hospitals and the nursing homes and everywhere need to be consistent about what they can and can't do, but they need a spokesperson to tell them that. PARTICIPANT 6

I think it's tricky, because wards will have carer students, EN students, RN students, students from all different unis and TAFEs and what not, so it can be difficult to define the role. PARTICIPANT 3

#### 5.3.3 Limited Preparedness of RNS for WPV‐University Support

Despite the many formal resources and processes in place via the University, many of the CFs suggested support for nursing students could be strengthened by improving their preparedness for the reality of WPV in the clinical environment. There was a perceived lack of education and training regarding WPV when in clinical placement.

I don't think there's anything to prepare them for it. There might be an occasional bit in topic about dementia and challenging behaviours or something like that, but I can't say I've come across a lecture or a tute I've had to give where I say right, one day a visitor might yell at you, this is what you do. I don't think there's anything… This could be a simulation, this could be role‐playing an aggressive situation in a safe space in a lab and getting them to do some basic de‐escalation, basic de‐escalation, not safety training. Because it's not particularly in the curriculum but we talk about it, don't block your exit, talk nice to people, those kinds of things, walk away when they're getting aggressive. PARTICIPANT 5

They need to take a more active role; not just being a poor cousin in the party because it feels like they give a lot of delegation to their clinical facilitators. more input from university would be grateful… because they are easy to take money from students. They implement a lot of stuff but when it comes to the real realisation and follow the policies it's a big gap. PARTICIPANT 4

I don't think there's any particular training or strategies in particular for—specific, just for nursing students. PARTICIPANT 3

CFs suggested universities incorporate WPV incidents and strategies during clinical orientation as part of the university curriculum as a combination of theory and practice sessions.

It's just in clinical orientation we need to talk more about it. Some part of our curriculum, it's something that can be in there so that nurses or future nurses, registered nurse can be aware because they're going into placement. It's not just about theory, they're doing the practice as well so they need to be aware of it and it needs to be discussed more… So, we do touch on that, so this is how you're professional, this is how you can be respectful, this is how you can build a rapport with your colleagues, with your senior nurses, with the doctors, with the physio. But we don't say this is what you need to do if a patient were to lash out at you. There's no, it's not really obvious where from the uni side of this. Whether that's what we do on clinical orientation, it's not something we've actually talked about. I said we go into the fact that they need to be professional and how to have a really good relationship with other members of the, like the staff members but I think it's lacking from that perspective when it comes to the uni side of things. PARTICIPANT 7

CFs believe that the university must support nursing students well before going into placements and, therefore, must be made aware of the strategies they should consider following the WPV incident.

I don't think it's probably talked about enough, within the university of going back to when I in place. And I don't know if the students are prepared enough knowing the risks when they're coming into a specialised area such as mental health or anything. PARTICIPANT 8

There's no implementation. Often what happens is students come back to university with a story about something that happened on placement that was challenging. It could be violent, bullying, aggressive from staff, visitors, patients. And then we're actually talking about it in the classroom as part of a debrief scenario. Have you thought about this, what factors are here, what would you do, what have you done? And you just wonder did these students get this support out there? Certainly, didn't get it before they went. PARTICIPANT 5

Although many of the issues and limitation of the support provided to RNS identified by CFs relate to Universities and RNS preparation, there were significant issues highlighted by CFs that clinical venues could address.

### 5.4 Clinical Venue: Support Actions and Limitations

#### 5.4.1 The Effects of Clinical Allocation by Venue Staff

The clinical venue is a complex environment with many competing agendas and priorities. When RNS arrive on to a ward or venue, they are allocated by the staff to specific areas or patients. It is this allocation that draws some comments from the CFs, as there are often inequities or inappropriate allocations that put ill‐equipped RNS in positions where WPV is a known frequent event. The shift manager or team leader allocates nursing students to experienced nurses individually based on recognising the personality of staff and patients; however, the allocation was not always well considered, and poor allocation of students to wards or areas had a significant detrimental effect on students physical, mental and emotional health, as these CFs described.

So definitely allocation, so where they're going to be allocated. And I think more care needs to be taken by the individual, the shift managers to allocate to a particular personality, or the patient they get paired with…. So, like in my organisation they say that student nurses shouldn't be looking, should be, you know supervised by someone looking after a ventilated patient or have dialysis or whatever. But the alternative to that is they get paired with a junior nurse who's looking after 2 patients and they get abandoned, and that's not okay. And that junior nurse doesn't have the life experience to be able to identify if something's going on with that student. PARTICIPANT 6

There are some facilities out there that are kind of notorious for not treating students very well. which universities have still sent students to, and then just kind of—yeah, it's a set‐up to failure, sometimes I feel. I've spoken to a few facilitators and a few, like topic coordinators and stuff, and they're like, oh yep, nup, that ward's notorious for it. PARTICIPANT 3

I remember a facilitated student who was really cooked up on this ward. So halfway through the placement she came to me, and she said I am just switching nursing all up, and I am just stopping the placement, it's not for me. The environment was very unsupportive, and I actually said Dana, we have probably a two‐hour conversation straight out. I said look you … something really and she was a third year student by the way, and I said look I have really invested so much time and everything, and so I said look what can be done, and I went and saw the big guns up on the ward and I actually found one nurse who was very responsible and she was very sensible, and she actually took her under her wing and that was it. Then I knew through other students she successfully completed the degree. PARTICIPANT 4

#### 5.4.2 Active Role and Support by Clinical Venues

Despite the many issues identified by the CFs, several CFs remarked on how the clinical venue staff were supportive of students by protecting them from exposure to WPV incidents, sometimes allowing for debriefing and enabling students to talk or be aware of their challenges during placement.

It doesn't happen all the time but they're quite proactive in protecting them from that exposure… I found that depending on the students themselves, they may have debriefed with the nurse that they were working with before I got to the situation or they might actually be waiting for me or would have given me a call to say I need to talk to you. PARTICIPANT 7

I find most of the allied staff to be extremely helpful toward our students. I think they may go in to bat a little bit more for our students, I don't know why that would be, maybe it's because you now that most of the time if they need a hand getting a patient out of bed, so physio 9 times out of 10 it's going to be the student that's going to do it. So, they don't want to get on the wrong foot with the student because then if they don't get the student, they might not get the staff member within a suitable time frame. I don't know if that's what it is, I'm not sure I still, I've not, I guess I've not investigated that. But at the same time, I think you know speech path, physios they're all really quite supportive of our students. PARTICIPANT 9

#### 5.4.3 Positive Ward Experiences

Improving nursing students' positive experience on the wards was considered necessary to retain them in the profession. CFs believed that by supporting and teaching the RNS to be professional, RNS learn skills that assist them throughout their nursing careers.

Have a positive experience on the ward might be enough. If you can match them with someone who won't create a problem, you might be able to reduce their experiences or at least prevent them from leaving their program. So, it's heart breaking the idea a student would leave after three years of commitment. PARTICIPANT 4

By being professional and doing your professional thing, and that way you know that you've done the right thing, even if that person hasn't. But that's a skill to learn, a skill to teach. PARTICIPANT 1

#### 5.4.4 Limitation of Support Following WPV Event at Clinical Venues

CFs found that support for nursing students following incidents of WPV was varied and at times limited, and that services in place were often inadequate to address issues of WPV against SRN. CFs mentioned the need for more support from venue managers following reports of WPV incidents, a need for onsite services, and more transparency when dealing with WPV.

I would say that the majority of the students I've spoken to have had some kind of debrief with the nurse when this aggression or challenging behaviour happens. So, in that sense, they've had the opportunity to debrief. However, do I think that it was done well and supported? Not always. Because these are staff members who are used to it and won't just fob off what students think, yeah, you'll be okay. But in saying that, when they come back to the university and share their horror stories of what they've been through, it doesn't sound like they've been supported well through some of these challenges they've had on placement around violence. PARTICIPANT 5

We don't have onsite facilities for counsellors and that on our hospital. It'd be good if every hospital had somebody there that's easily accessible that they should be able to go to talk to immediately. PARTICIPANT 8

The organisation that they're doing the placement in, especially if it's a smaller organisation, will try and hide problems. PARTICIPANT 6

At the end of the day… employer assisted program I think it's quite useless because they don't address things because at the end of the day it comes to manager and the manager would say oh, he reports to me, oh bloody hell. So, I will give him a hard time. So, it comes back to you as an employer. PARTICIPANT 4

## 6 Discussion

The CFs who participated in the interviews identified a range of supports students have access to following WPV incidents, but also acknowledged many limitations of that support and opportunities for that support to be improved. Hospital staff, including nurses, shift leaders and allied health professionals, were key to providing students with support and a nurturing environment, going “into bat” for them when they are struggling. CFs indicated that thoughtful allocation of nursing students, positive interactions with hospital staff, and transparency in accessing support services protected students from WPV and empowered students to report WPV incidents. CFs and other university staff, including administrators and counsellors, were also integral to assisting students, preparing them for the risk of WPV they may be exposed to in health care settings, incorporating strategies to prevent or minimise WPV within the curriculum, and providing support following incidents of WPV, such as helping students find a new placement. Through interviews, the CFs stated that universities can better support students by clarifying the capacities of students across a range of programs, so nurses can be clear on what roles and responsibilities are within the scope of the students they are supervising. Family and friends were reported to be the most important source of support for students, from the perspective of the CFs.

There were limitations to the support provided in clinical settings and by university staff. CFs in these interviews felt universities could have a more active role in preparing students for the risk of WPV in clinical settings and should have greater control over the treatment of students on placement. Additionally, more needed to be done within universities to support students following a WPV incident.

CFs related timely and meaningful support for students to students' capacity to cope with incidents of WPV and successful completion of their undergraduate program. Nursing students need a safe and supportive environment to thrive during placements, but many registered nurses responsible for supervision feel unprepared and unsupported in this role (Newton et al. 2018). Additionally, in the Australian context, the proportion of international nursing students is growing. As of 2018, around a fifth of commencing nursing and midwifery students are international (17.9%) (Department of Health and Aged Care 2020). International students require more support from supervising nurses compared to domestic students to accommodate for cultural differences, language barriers and mismatched expectations around learning styles, and nurses indicate they get little help from universities on how to best support international nursing students (Newton et al. 2018). The provision of support from a range of sources is key to the success of nursing students; clinical coaches, peer mentorship, and support from faculty have been shown to decrease attrition rates and reduce student levels of stress (Smith‐Wacholz et al. 2019). Support from family and friends was also important, fostering resilience in nursing students and improving retention (Thomas and Revell 2016).

Support from hospital staff was associated with positive learning experiences, improved clinical practice and increased confidence, as reported by the facilitators in our cohort. While much of the extant evidence in the international literature focuses on negative interactions with staff, some studies have reported instances where nursing students share how support provided by hospital staff has benefited their learning (Dafny et al. 2023). Hospital systems are such that supports for students from both the structural nature of healthcare and nursing staff are lacking. Nursing students on placement are supernumerary to the staff count and should be seen as such in all cases. This idea then allows for students to ideally experience the role of the RN without the responsibility of a workload, allowing for progression with both skills and the foundational knowledge required to later become staff.

Registered nurses sit within a hierarchal system, ranked by years of experience and clinical title. As such not all nurses may feel safe and empowered within their roles as healthcare professionals. Staff who are feeling vulnerable are less likely to be able to support and nurture a nursing student when they themselves feel at risk within the clinical setting (Newton et al. 2018). The ‘nurses eat their young’ mantra has added a complication to what supports can be seen. Nursing culture in Australia has created a culture in which complaining about WPV or reporting it may not only be discouraged but also seen as a threat to the hierarchical system that is in place. Although healthcare systems do have policies in place regarding bullying and WPV the reporting system is not always transparent and easy to access. When adding the perceived threat of loss of placement that some nursing students report then it is easy to see the discomfort that can be felt with addressing this WPV (Dafny et al. 2023; Johnston et al. 2024).

Support from CFs and other university staff helps students to better prepare for their placements and future employment. Internationally, a recent scoping review found the role of the CF is considered ‘pivotal’ for enhancing nursing students clinical learning (Rojo et al. 2020). Australian nursing students have reported that university‐based CFs play an important role in their learning and the time and support facilitators can provide are highly valued (Sweet and Broadbent 2017). The facilitators in our cohort agreed with a recent Australian study investigating the experiences of CFs, which found facilitators interpreted their role to include developing student knowledge and facilitating successful clinical experiences, but also acting as a counsellor and protector for vulnerable and stressed students while on placement (Ryan and McAllister 2019).

While the role of CFs is considered essential for enhancing student success in placement, lack of role clarity, limited support and training for CFs, along with the cost‐cutting measures of universities results in facilitators who are under pressure and caught between the expectations of universities and clinical settings (Ryan and McAllister 2021; Hughes et al. 2023). This disempowered position could be addressed if the universities have a good connection with the workplace and have a clear direct line to the management to address any potential issues that arise or with a zero‐tolerance policy regarding WPV (Tee et al. 2016). CFs, as employees of the University, are seen to hold the primary responsibility for the safety of a clinical setting and the experience of the students (Warshawski 2021). This is supported by Tee and Valiee (2020), stating that addressing WPV is the responsibility of the university staff rather than being a role that is supported by the clinicians, also explaining that the hierarchy of nursing culture and the power imbalance it creates can impact WPV for students. Debriefing and competent counselling are important following an episode of violence (Dafny and Muller 2021), but the time to complete forms and report to internal structures is not always implemented. Understanding this, as the experience of the continuing staff, it is easy to see the hierarchical trickle‐down effect upon students on placement when they experience WPV.

### 6.1 Strengths and Limitations of the Research

The study investigated CFs' perspectives on how they can support nursing students following experiences of WPV during their clinical placements. However, its applicability is limited due to the participation of CFs exclusively from one institution in South Australia. The specific institutional nuances and arrangements with clinical facilities may affect the outcomes, potentially making the findings institution‐specific. Therefore, replication of the study in other institutions is recommended.

### 6.2 Recommendations for Future Research

The findings of this study highlight the need for increased preparation of RNS prior to clinical placements; thus, further robust research needs to be conducted on how best to prepare RNS for the rigours of WPV in the clinical venue. Furthermore, understanding which support tools and strategies are most valued to support victims of WPV also needs to be considered.

### 6.3 Implications for Policy and Practice

Strong collaborations and established relationships between universities and clinical facilities, along with clear guidelines and direct lines to address potential issues with a zero‐tolerance policy regarding WPV and support for CFs and nursing students, could provide a safer environment and better experience to promote nursing student learning outcomes. WPV should be addressed as a multi‐organisation approach based on collaboration, trust and respect; when both universities and healthcare providers take a more active role in preparing nursing students and supporting them within the clinical setting, it could have long‐reaching impacts on the recruitment and retention of the nursing profession, the healthcare industry and the health outcomes of anyone cared for within this system.

WPV negatively affects nurses' and nursing students' well‐being, satisfaction, retention and recruitment. Nursing students, especially students with cultural and linguistic diversity, have further consequences of WPV due to language barriers and mismatched expectations around learning styles and, therefore, require more support and mentoring from CFs, universities and clinical facilities. The role of the CF is critical for enhancing nursing students' clinical learning, acting as a counsellor and protector for vulnerable and stressed students while on placement. However, to best facilitate a safe and healthy learning experience, they must be provided with the appropriate education and tools to support students in a challenging environment, especially casual facilitators who see themselves as visitors in new environments.

## 7 Conclusion

Hospital systems encounter significant challenges when it comes to adequately supporting nursing students during their clinical placements. These challenges arise from both structural limitations and the hierarchical nature of nursing roles. Registered nurses, despite their experience and clinical titles, may not always feel safe or empowered in their roles. Consequently, they might struggle to effectively support and nurture nursing students, especially when they themselves feel vulnerable.

CFs, who play a critical role in ensuring student safety during placements, often lack clear direction and proper education to provide appropriate support to nursing students after WPV. Their responsibilities extend beyond mere supervision; they must provide unwavering support to nursing students throughout their clinical placement, particularly in navigating challenges such as WPV. The impact of such incidents on both students and facilitators is profound, affecting their well‐being in both the short and long term. When CFs perceive themselves as ill‐equipped or powerless to assist students in such situations, it can jeopardise the students' well‐being and their ability to meet clinical placement objectives.

Innovative strategies for support provision are essential during these times, necessitating a collaborative effort between the clinical facility and the university. To address these challenges, universities should establish strong connections with clinical facilities and provide clear guidelines for facilitators. The responsibility for addressing WPV primarily lies with university staff, as clinicians may not consistently support this role. While debriefing and counselling are essential after WPV incidents, administrative barriers can sometimes delay reporting. In summary, addressing WPV requires systemic changes, better support for CFs and a steadfast commitment to student safety.

## Author Contributions

H.A.D. designed the study; all authors acquired, analysed the data and wrote the manuscript.

## Conflicts of Interest

The authors declare no conflicts of interest.

## Supporting information


Data S1.

## Data Availability

The data that support the findings of this study are available from the corresponding author upon reasonable request.
