# Supplementary material for: Stakeholders' Actions, Responsibility and Limitations in Support of Nursing Students Experiencing Workplace Violence During Clinical Placement: The Clinical Facilitators View
Source: J Clin Nurs. 2025 Feb 19;34(6):2348–59. doi: 10.1111/jocn.17706 (PMC12125530; doi:10.1111/jocn.17706)
Supplement: Supplementary file 1 — Data S1. [file JOCN-34-2348-s001.docx]

**Consolidated criteria for reporting qualitative studies (COREQ): 32-item checklist based on Tong, Sainsbury, and Craig (Updated March 2015)**

**Domain 1: Research team and reﬂexivity**

**Personal Characteristics**

1. Interviewer/facilitator- Which author/s conducted the interview or focus group? Dr Hila Dafny conducted the interviews reported on in this manuscript. Page 3
2. Credentials- What were the researcher’s credentials? RN, BSN, MPH PhD Title page
3. Occupation-What was their occupation at the time of the study? Dr Dafny is a Senior Lecturer at Flinders University of South Australia Title Page
4. Gender-Was the researcher male or female? Female Title Page
5. Experience and training What experience or training did the researcher have? Dr Dafny is a well-recognised researcher with many completed research projects and publications of the findings of her research. Participant Information Sheet

**Relationship with participants**

1. The participants of the study were Clinical Facilitators who are appointed by the Flinders University South Australia (FUSA) to support the Nursing students in the clinical environment. Research team members who had worked closely with the clinical facilitators were precluded from the interview component of the research, as this was identified as a potential conflict of interest. Page 3
2. Participant knowledge of the interviewer-What did the participants know about the researcher? All participants were provided with a detailed information sheet that clearly identified the aims, purpose and methods of the research and who the researchers were. Participant Information Sheet
3. Interviewer characteristics-What characteristics were reported about the interviewer/facilitator? The interviewer was an experienced interviewer who has published a number of primary research articles as first author and is a Senior lecturer at the Research facility (FUSA). The Clinical facilitators (participants) were RN’s who either worked in or were allocated specific workplace sites where they had the responsibility of supporting the student RN’s clinical experience. Participant Information Sheet

**Domain 2: study design**

**Theoretical framework**

1. Methodological orientation and Theory-What methodological orientation was stated to underpin the study? This research used an established phenomenological research approach that explores the clinical facilitators "lived experience" of WPV. Data collection methods used in this study were semi structured interviews. Thematic analysis of the participant interview transcriptions are study findings, where commonalities of reported experiences are aggregated. Page 3

**Participant selection**

1. Sampling-How were participants selected? Clinical Facilitators who are responsible for the WIL of student RN’s of FUSA were purposively sampled as having experience of or witnessing WPV. Page 3
2. Method of approach-How were participants approached? Participants were recruited using email, printed and online invitations posted within each departments bulletin board and the FUSA email system. Study Recruitment Flyer
3. Sample size-How many participants were in the study? Eleven Clinical Facilitators were included in the study. Page 3
4. Non-participation-How many people refused to participate or dropped out? Reasons? No CF’s dropped out from the study after initial contact and interest in participation. Documentation provided to the CF’s emphasised that participation was entirely voluntary. Participant Information Sheet

**Setting**

1. Setting of data collection-Where was the data collected? Participants who agreed to be interviewed chose their preferred location, face-to-face or via Microsoft Teams (online), date and time. Page 3
2. Presence of non-participants-Was anyone else present besides the participants and researchers? No

Description of sample-What are the important characteristics of the sample? The eleven Clinical Facilitators, the participants of this study were employed in a wide range of clinical environments from acute care to mental health care. They had a wide range of career lengths and were mostly female. Page 3

**Data collection**

1. Interview guide-Were questions, prompts, guides provided by the authors? Participants of the study were provided an information sheet about the study prior to the interviews. The semi structured interview guide was provided to the participant prior to the interview. Demographic information was collected during the initial phase of the interview. This information established the context and experiences of the CF’s WIL. An outline of the questions asked in the interview included: Have you ever experienced or witnessed workplace violence while you were in clinical placement with nursing students? Would you like to share your experience? Do you think that nursing students are provided support after violent incidents? What kind of support? By whom? What do you suggest in order to reduce or avoid violence toward nursing students? as a clinical facilitator? University? Your workplace/institution?  Further open-ended questions encouraged narrative answers from the participants. When necessary, probing and prompts such: “What do you mean by…?” or “Please, tell me more about…” were used to deepen the richness of the data collected. Interview Guide and pages 3-4.
2. Repeat interviews-Were repeat interviews carried out? No.
3. Audio/visual recording-Did the research use audio or visual recording to collect the data? All interviews were conducted via face-to-face or via Microsoft Teams (online) Page 3
4. Field notes-Were ﬁeld notes made during and/or after the interview or focus group? Field notes were made during and after the interviews.
5. Duration-What was the duration of the interviews or focus group? Interviews were each up to 60 minutes long. Page 3
6. Data saturation-Was data saturation discussed? During the conceptual and design phase of the study planning, the sample size was discussed, and the concept of data saturation was identified as the most appropriate end point of data collection. Thus, data continued to be collected until no new information or themes appeared from the interviewed participants and the data became repetitive. Page 4
7. Transcripts returned-Were transcripts returned to participants for comment and/or correction? The transcripts have not been returned to the participants for comment due to the recording were deleted after transcription, and pseudonyms were used in transcripts to ensure anonymity and confidentiality. Page 4.

**Domain 3: analysis and ﬁndings**

**Data analysis**

1. Number of data coders-How many data coders coded the data? Two of the research team were responsible for the initial coding with the results circulated to all members of the team for comment and review. Page 4.
2. Description of the coding tree-Did authors provide a description of the coding tree? The initial coding tree with all parent and child nodes was made available to the team for comment. As analysis proceeded the child themes were aggregated under specific and appropriate parent themes. Page 4 and Table 1 (*Themes and subthemes identified through thematic analysis*).
3. Derivation of themes-Were themes identiﬁed in advance or derived from the data? All themes and subthemes of the findings of this study were derived directly from the data and where patterns, similarities and differences were identified. Table 1.
4. Software-What software, if applicable, was used to manage the data? The first stage of data analysis required deep immersion in the data via the reading and re reading of the transcriptions. Once the researchers responsible for the first level of analysis felt deeply immersed in the data it was imported into the soft ware NVivo 12 where text segments were coded to emerging child and parent themes. Page 4.
5. Participant checking-Did participants provide feedback on the ﬁndings? Participant checking was not employed in the analysis of this data. The data analysis via multiple researchers ensured triangulation of analysis and supported trustworthy findings. Page 4.

**Reporting**

1. Quotations presented-Were participant quotations presented to illustrate the themes / ﬁndings? Yes, in the final report of the findings of the study and consistent with reporting of qualitative data quotes from participants were used extensively to illustrate and support the findings. Pages 5-11.
2. Data and ﬁndings consistent-Was there consistency between the data presented and the ﬁndings? Yes, the data presented as themes was strongly supported by the exemplar statements of the participants displaying consistency between data and findings. Pages 5-11.
3. Clarity of major themes-Were major themes clearly presented in the ﬁndings? Major themes with subthemes were clearly presented in the findings with tables used to display the strength of findings. Pages 5-11.
4. Clarity of minor themes-Is there a description of diverse cases or discussion of minor themes? Similarities and differences that occurred in the data prompted discussion and suggested explanations for the diversity were made in the findings and discussion. Pages 5-11.

**References:** Tong, A., Sainsbury, P., & Craig, J. (2015). Updated; Consolidated criteria for reporting qualitative research (COREQ): a 32-item checklist for interviews and focus groups. *International Journal for Quality in Health Care, 19*(6), 349-357. Retrieved from https://doi.org/10.1093/intqhc/mzm042. doi:10.1093/intqhc/mzm042
